# TSG From *Polygonum multiflorum* Thunb. Attenuates Experimental Premature Ovarian Insufficiency in Association With Reduced ZBP1/PANoptosis Signaling

**DOI:** 10.1002/fsn3.72347

**Published:** 2026-09-08

**Authors:** Jinhong Li, Tiao Xie, Chang Lin, Danhong Peng, Qin Shi, Can Zhu

**Affiliations:** ^1^ College of Basic Chinese Medicine Guizhou University of Traditional Chinese Medicine Guiyang Guizhou China; ^2^ Shizhen College of Guizhou University of Traditional Chinese Medicine Guiyang Guizhou China; ^3^ College of Pharmacy Guizhou University of Traditional Chinese Medicine Guiyang Guizhou China; ^4^ College of Basic Medicine Guizhou University of Traditional Chinese Medicine Guiyang Guizhou China

**Keywords:** 2,3,5,4′‐tetrahydroxystilbene‐2‐O‐β‐D‐glucoside, PANoptosis, PANoptosome, premature ovarian insufficiency, Z‐DNA binding protein 1

## Abstract

*Polygonum multiflorum* Thunb. is a widely consumed functional food and dietary supplement in East Asia. However, whether its bioactive component, 2,3,5,4′‐tetrahydroxystilbene‐2‐O‐β‐D‐glucoside (TSG), can alleviate premature ovarian insufficiency (POI) remains unknown. This study aimed to investigate whether TSG administration alleviates POI in association with suppression of ZBP1/PANoptosis signaling. A tripterygium glycoside‐induced POI rat model was treated with TSG via intraperitoneal injection. Ovarian function was assessed via estrous cycles, hormone assays (FSH, E_2_, AMH), and histopathology. The formation of the ZBP1/PANoptosome and key executioners of PANoptosis were analyzed by immunofluorescence and Western blot. In triptolide‐injured KGN granulosa cells, TSG's effects were measured by assessing cell viability, apoptosis, lactate dehydrogenase (LDH) release, and PANoptosis activation with or without ZBP1 overexpression. TSG significantly restored estrous cyclicity, improved hormone profiles, and preserved follicular reserve in POI rats. At the molecular level, TSG was associated with reduced ZBP1/PANoptosome formation and downstream executioners (e.g., cleaved Caspase‐3, N‐GSDMD, p‐MLKL). In cells, TSG mitigated cellular injury*.* Crucially, while ZBP1 overexpression aggravated damage and amplified PANoptosis signaling, TSG co‐treatment significantly reversed these effects, indicating that ZBP1/PANoptosis signaling may be associated with the protective effects of TSG against POI. In conclusion, TSG mitigates POI in association with the suppression of ZBP1/PANoptosis, thereby enhancing the mechanistic understanding of POI pathogenesis and positioning TSG as a candidate for further investigation.

## Graphical abstract

This study shows that TSG, the major bioactive constituent of *Polygonum multiflorum* Thunb., ameliorates POI in association with suppression of ZBP1‐driven PANoptosis. In a POI rat model, TSG restored estrous cyclicity, improved hormone profiles, and preserved follicular reserve. Mechanistically, TSG reduced ZBP1 expression, inhibited ZBP1‐PANoptosome assembly, and downregulated downstream effectors of PANoptosis. In KGN cells, ZBP1 overexpression exacerbated injury and PANoptosis signaling, effects reversed by TSG. These findings indicate that TSG mitigates POI in association with the suppression of ZBP1/PANoptosis, supporting its further investigation in POI management.

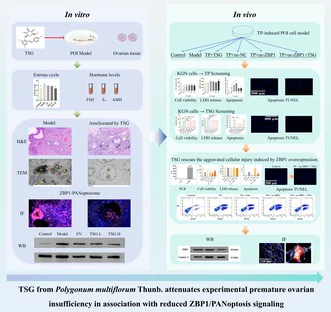

**Abbreviations**
AMH: anti‐Müllerian hormone
ASC: apoptosis‐associated speck‐like protein containing a CARD
c‐Caspase‐3: cleaved‐Caspase 3
CCK‐8: cell counting kit‐8
DAPI: 4′,6‐Diamidino‐2‐Phenylindole
E_2_
: estradiol
EV: estradiol valerate
FSH: follicle‐stimulating hormone
GAPDH: glyceraldehyde‐3‐phosphate dehydrogenase
GSDMD: gasdermin D
GSDME: gasdermin E
LDH: lactate dehydrogenase
MLKL: mixed lineage kinase domain‐like protein
oe‐NC: negative control for overexpression
oe‐ZBP1: overexpression of Z‐DNA binding protein 1
POI: premature ovarian insufficiency
RIPK3: receptor‐interacting protein kinase 3
TEM: transmission electron microscopy
TP: triptolide
TSG: 2,3,5,4′‐tetrahydroxystilbene‐2‐O‐β‐D‐glucoside
TUNEL: terminal deoxynucleotidyl transferase dUTP nick end labeling
ZBP1: Z‐DNA binding protein 1

## 1 Introduction

Premature ovarian insufficiency (POI) refers to the loss of ovarian function before the age of 40, typically manifested by menstrual disturbances such as oligomenorrhea or amenorrhea, elevated gonadotropin levels, and clinical signs of estrogen deficiency. It not only leads to infertility and perimenopausal symptoms but also increases the risk of long‐term complications, including osteoporosis, cardiovascular diseases, and cognitive dysfunction (Stuenkel and Gompel 2023). With a global prevalence of approximately 3.5% ~ 3.7% and a rising trend towards earlier onset, POI poses a substantial threat to women's health, family well‐being, and societal demographic stability (Panay et al. 2024). Current management is primarily based on hormone replacement therapy and assisted reproductive technologies, which offer symptomatic relief but fail to address the underlying etiology and may raise risks such as venous thromboembolism and hormone‐dependent malignancies with long‐term use (Poggio et al. 2022; Skeith et al. 2021). Therefore, a deeper understanding of POI pathogenesis and the development of effective preventive and therapeutic strategies remain a critical priority in reproductive endocrinology.

The etiology of POI involves immune, genetic, iatrogenic, and environmental contributors, yet over half of cases remain idiopathic. Aberrant activation of programmed cell death (PCD) represents a key pathological event that disrupts ovarian homeostasis, drives excessive follicle atresia, and contributes to POI progression (Stringer et al. 2023). PANoptosis is an inflammatory PCD mode recently defined by the concurrent activation of pyroptosis, apoptosis, and necroptosis, with crosstalk among these pathways (Lee et al. 2021; Wang and Kanneganti 2021). This process is controlled by a multiprotein complex known as the PANoptosome, which is formed in a context‐dependent manner from core molecular components of all three cell death pathways (Christgen et al. 2022; Lee et al. 2021). Z‐DNA binding protein 1 (ZBP1), an innate immune sensor, acts as a critical upstream initiator. Upon activation by specific triggers (e.g., pathogens or cellular stress), ZBP1 initiates PANoptosome formation, leading to the activation of downstream executioners, including gasdermin D/E (GSDMD/E), caspase‐3/7, and mixed lineage kinase domain‐like protein (MLKL), ultimately leading to lytic cell death (Kesavardhana et al. 2020; Samir et al. 2020; Wang et al. 2024; Zhu, Yao, et al. 2024). Although emerging evidence has implicated PANoptosis in ovarian dysfunction (Bai et al. 2025; Cui et al. 2024), and our previous study suggested that granulosa cell PANoptosis contributes to excessive follicular atresia in POI (Zhu, Li, Li, et al. 2025), the precise function of ZBP1 as a key regulator of PANoptosis in the pathogenesis of POI has yet to be determined.

The dried root of *Polygonum multiflorum* Thunb. is a widely consumed functional food and dietary supplement in East Asia, traditionally used to promote health and address aging‐related conditions (Tian et al. 2025; National Pharmacopoeia Commission 2025). According to traditional Chinese medicine theory, it is considered a kidney‐tonifying herb, as “the kidney stores essence and governs reproduction.” In this context, kidney deficiency is considered a core pathogenesis of POI, guiding treatment principles focused on kidney tonification (Tian et al. 2025). Its major bioactive component, 2,3,5,4′‐tetrahydroxystilbene‐2‐O‐β‐D‐glucoside (TSG), exhibits notable anti‐aging, antioxidant, anti‐inflammatory, and immunomodulatory properties (Charoensaensuk et al. 2022; Zhu, Li, Li, et al. 2025). Prior research has indicated that TSG postpones senescence triggered by oxidative stress in Leydig cells and maintains ovarian function in aging mice (Li et al. 2021; Lin et al. 2022). Our earlier work suggested that the water extract of this herb may be associated with modulation of serum sex hormone levels and with a slower decline of ovarian function in rats (Zhu et al. 2018). In addition, both the aqueous extract and isolated TSG enhanced uterine development and upregulated estrogen receptor expression in immature mice, supporting their phytoestrogen‐like activity (Zhu et al. 2019, 2020). Furthermore, our study showed that an extract of *Polygonum multiflorum* Thunb. suppresses PANoptosis in rat granulosa cells, thereby limiting excessive follicular atresia and decelerating the progression of POI (Zhu, Li, Li, et al. 2025). However, whether its principal active constituent TSG confers similar protection through inhibition of ZBP1/PANoptosis remains unclear.

Using in vivo and in vitro experiments, this study investigated whether TSG is associated with amelioration of POI through mechanisms that may involve the modulation of ZBP1/PANoptosis (a novel coordinated cell death pathway involving pyroptosis, apoptosis, and necroptosis). This work is expected to provide novel mechanistic understanding of POI pathogenesis and to establish an experimental basis for developing TSG‐based strategies associated with PANoptosis modulation in POI.

## 2 Materials and Methods

### 2.1 Chemicals and Reagents

TSG (Cat# T874838, 99% purity) and triptolide (TP, Cat# T819208, 99% purity) were obtained from Shanghai Macklin Biochemical Co. Ltd. (Shanghai, China). Estradiol valerate (EV; Cat# 821A) was purchased from Bayer Healthcare Co. Ltd. (Leverkusen, Germany). Tripterygium glycosides (Cat# 20240701) were supplied by Hunan Qianjin Xieli Pharmaceutical Co. Ltd. (Hunan, China). Commercial assay kits were used: follicle‐stimulating hormone (FSH; Cat# E‐OSEL‐R0001, Elabscience, China), estradiol (E_2_; Cat# E‐EL‐R0391, Elabscience, China), anti‐Müllerian hormone (AMH; Cat# E‐EL‐R3022, Elabscience, China), cell counting kit‐8 (CCK‐8; Cat# BS350B, Biosharp, China), terminal deoxynucleotidyl transferase dUTP nick‐end labeling (TUNEL; Cat# E‐CK‐A325, Elabscience, China), and lactate dehydrogenase (LDH; Cat# E‐BC‐K766‐M, Elabscience, China). The following primary antibodies were used in this study: anti‐ZBP1 (Cat# A13899, ABclonal, China), anti‐apoptosis‐associated speck‐like protein containing a CARD (ASC; Cat# DF6304, Affinity Biosciences, USA), anti‐caspase‐8 (Cat# AF6442, Affinity Biosciences, USA), anti‐caspase‐3 (Cat# A2156, ABclonal, China), anti‐cleaved caspase‐3 (Cat# 68773–1‐lg, Proteintech, USA), anti‐GSDMD/N‐terminal GSDMD (Cat# A24476, ABclonal, China), anti‐GSDME (Cat# A7432, ABclonal, China), anti‐MLKL (Cat# A17312, ABclonal, China), anti‐phospho‐MLKL (Cat# AF7420, Affinity Biosciences, USA), anti–receptor‐interacting protein kinase 3 (RIPK3; Cat# A5431, ABclonal, China), and anti‐GAPDH (Cat# AF7021, Affinity Biosciences, USA).

### 2.2 POI Rat Model and Treatment

Female specific pathogen‐free Sprague–Dawley rats (8 weeks old, 200–220 g) were purchased from Henan Sikebas Biotechnology Co. Ltd. (Henan, China) and maintained under standard housing conditions at the Laboratory Animal Research Center of Guizhou University of Traditional Chinese Medicine. All animal experiments complied with internationally accepted guidelines for laboratory animal welfare and were approved by the Animal Ethics Committee of Guizhou University of Traditional Chinese Medicine (Approval No. 20241013001). This study was conducted in accordance with the ARRIVE guidelines (https://arriveguidelines.org).

Rats were randomly assigned to five groups (*n* = 6 each) using a computer‐generated randomization sequence: control, model, estradiol valerate (EV; positive control), TSG low‐dose (TSG‐L), and TSG high‐dose (TSG‐H). The investigators responsible for group assignment and outcome assessment were masked to group allocation throughout the experiments and data analysis. All drugs were prepared in normal saline prior to administration, using standardized volumes of 10 mL·kg^−1^ for gavage and 5 mL·kg^−1^ body weight for intraperitoneal injection. Except for the control group, all other groups received daily oral gavage of tripterygium glycosides at 75 mg·kg^−1^ each morning to induce the POI model (Chen et al. 2025; Xiao et al. 2026) while controls were given saline. In the afternoon, the control and model groups were given intraperitoneal injections of saline, whereas the EV group was treated intraperitoneally with estradiol valerate at 0.09 mg·kg^−1^ (Hou et al. 2025), and the TSG‐L and TSG‐H groups received TSG (10 and 40 mg·kg^−1^, i.p., respectively) (Lin et al. 2022; Liu, Chen, et al. 2024). Estrous cycles were monitored daily by vaginal smear cytology (Shreya et al. 2025).

After 28 consecutive days of treatment, rats were anesthetized by intraperitoneal injection of sodium pentobarbital. Blood and ovaries were collected, and the rats were then euthanized by cervical dislocation. In the experiment, body weight was recorded weekly, and food intake as well as general behavior were monitored daily; no abnormal behaviors or adverse effects were observed. Humane endpoints were predefined as body weight loss exceeding 20%, severe lethargy, or inability to reach food or water, and no animals reached these criteria during the study. The ovarian index was determined as (ovarian wet weight/body weight) × 100%. TSG was administered concurrently with the POI‐inducing agent, making this a preventive/co‐treatment regimen.

### 2.3 Hematoxylin and Eosin (HE) Staining and Follicle Count

Ovarian tissue specimens (*n* = 6 per group) were collected, fixed in 4% paraformaldehyde, and embedded in paraffin. Subsequently, serial sections of 4 μm thickness were prepared and stained with hematoxylin and eosin. Three consecutive sections from the maximal cross‐section of each ovary were chosen, and follicles were categorized and enumerated based on established morphological criteria (Uslu et al. 2017) as follows: primordial follicles, identified by an oocyte surrounded by a single stratum of flattened granulosa cells; primary follicles, distinguished by an oocyte invested by one or more layers of cuboidal or columnar granulosa cells and surrounded by a distinct zona pellucida; secondary follicles, defined as further developed primary follicles showing fluid‐filled antral spaces among granulosa cells; and atretic follicles, characterized by degeneration of the oocyte and granulosa cells along with a shrunken zona pellucida (Li and Li 2015). Follicles were counted only when a clearly visible oocyte nucleus was present.

### 2.4 Serum Hormone Analysis

To obtain serum, whole blood samples (*n* = 6) were first clotted (1 h, room temperature) and then centrifuged (2000 rpm, 10 min). Subsequently, serum levels of AMH, FSH, and E_2_ were measured via commercial ELISA kits following the provided guidelines. Absorbance at 450 nm was recorded, and hormone concentrations, with six replicates per sample, were derived from the standard curve.

### 2.5 Transmission Electron Microscopy (TEM)

For ultrastructural analysis, ovarian cortical samples were immersed in a primary fixative containing 2% paraformaldehyde and 2.5% glutaraldehyde. Post‐fixation was performed using 1% osmium tetroxide. The tissues were then dehydrated in a graded series of ethanol, cleared with acetone, and processed for progressive resin infiltration. The samples were pre‐polymerized overnight at 37°C, and infiltration and polymerization were completed at 40°C and 60°C, respectively. Toluidine blue staining of semi‐thin sections was used to identify follicular granulosa cells, guiding the subsequent preparation of 90 nm ultrathin sections. Following double‐staining with uranyl acetate and lead citrate, the prepared sections were visualized under a transmission electron microscope.

### 2.6 Cell Culture and Transfection

KGN cells were purchased from Procell Life Science & Technology Co. Ltd. (Cat# CL‐0603, China) and authenticated by the supplier via STR genotyping. All cells tested negative for mycoplasma contamination. KGN cells were propagated in growth medium consisting of DMEM/F12 supplemented with 10% fetal bovine serum and 1% penicillin–streptomycin and incubated at 37°C with 5% CO_2_.

ZBP1 overexpression plasmid was designed and synthesized by GenePharma Co. Ltd. (Shanghai, China). Logarithmically growing KGN cells were seeded in 24‐well plates and assigned to two groups (six replicates each): negative control for overexpression (oe‐NC) and overexpression of ZBP1 (oe‐ZBP1). Transfection was performed using Lipofectamine 2000 (Cat# 11668027, ThermoFisher, USA) according to the manufacturer's instructions. Plasmids were mixed with transfection reagents in Opti‐MEM for complex formation, followed by addition to the culture medium. After 5 h of transfection, the medium was replaced with fresh complete medium. Cells were harvested 48 h post‐transfection, and transfection efficiency was verified by quantitative polymerase chain reaction (qPCR) assay. The relative mRNA expression level of ZBP1 was calculated using the 2^−ΔΔCt^ method with β‐actin as the internal reference. The primers were synthesized by Sangon Biotech Co. Ltd. (Shanghai, China): β‐actin: (Forward) 5′‐AATCTGGCACCACACCTTCTACAA‐3′, (Reverse) 5′‐GGATAGCACAGCCTGGATAGCAA‐3′; ZBP1: (Forward) 5′‐AGAATCCTGCAGGTGCTGAC‐3′, (Reverse) 5′‐GGACTTGGTTGAGCTCCCTC‐3′.

### 2.7 Establishment of Cellular POI Model and TSG Dose Screening

Triptolide (TP) is often used to induce cellular POI model (Xiao et al. 2026; Gao et al. 2025; Zhu, Zhang, et al. 2024). Log‐phase KGN cells were plated in 96‐well plates and distributed across six groups (3–6 replicates per group): TP concentrations of 0, 0.5, 1, 5, 10, and 50 μM. After 48 h of incubation, cell viability (CCK‐8 assay), lytic death (LDH release assay), and apoptosis (TUNEL assay) were assessed following the manufacturers' instructions. Based on these results, the optimal TP concentration for inducing the POI cell model was determined for subsequent experiments.

For TSG dose screening, log‐phase KGN cells were divided into six groups (3–6 replicates each): control, POI model (TP), TP + TSG (1, 10, 50, and 100 μM concentrations of TSG, respectively). After 24 h or 48 h of incubation, cell viability, lytic death, and apoptosis were assessed. A TSG concentration that consistently showed significant cytoprotection across these assays was selected for subsequent experiments.

### 2.8 Cell Viability Assay

Cells were plated and allocated into six experimental groups (six wells per group): control, model (TP), TP + TSG, TP + oe‐NC, TP + oe‐ZBP1, and TP + oe‐ZBP1 + TSG. At 48 h post‐treatment, each well received 10 μL of CCK‐8 solution, after which the plates were incubated at 37°C for 4 h. Cell viability was then evaluated by measuring the absorbance with a microplate reader.

### 2.9 Lactate Dehydrogenase (LDH) Release Assay

Cells were divided into five groups (three replicates each): control, model (TP), TP + TSG, TP + oe‐ZBP1, TP + oe‐ZBP1 + TSG. Following 48 h of treatment, culture medium was collected and subjected to centrifugation (2000 rpm, 10 min) to pellet cellular debris. The cleared supernatants were then incubated with the LDH reaction mixture in the dark, after which LDH release was quantified by measuring absorbance with a microplate reader.

### 2.10 TUNEL Staining

Cells were grouped and treated as described in the LDH assay (five groups, three replicates each). After 48 h of incubation, cells were fixed, cytospun onto glass slides, and then treated successively with TdT equilibration buffer followed by TUNEL reaction mixture. Slides were mounted with anti‐fade mounting medium, and nuclei were subsequently counterstained with DAPI. Images from three random fields per slide were captured under a CX40 fluorescence microscope (SOPTOP, China), and the percentage of TUNEL‐positive (apoptotic) cells was quantified using Image‐pro plus 6.0 software (Media Cybernetics, USA).

### 2.11 Annexin V‐FITC/PI Assay

Cells were grouped and treated as described in the LDH assay (five groups, three replicates each). After 48 h of treatment, cells were collected, centrifuged, and resuspended. Cells were then stained with Annexin V‐FITC and propidium iodide (PI), followed by a 5 min incubation at room temperature in the dark. Apoptotic and necrotic cell populations were analyzed using a CytoFLEX flow cytometer (Beckman Coulter, USA) and CytExpert software (version 2.4.0.28).

### 2.12 Immunofluorescence Staining

Ovarian paraffin sections (*n* = 3) underwent deparaffinization, rehydration, and antigen retrieval, followed by quenching of endogenous peroxidase and blocking of non‐specific binding. Sections were then incubated overnight at 4°C with primary antibodies: anti‐Caspase‐8 (1:200), anti‐ASC (1:200), and anti‐RIPK3 (1:100). Following washes, the sections were incubated with appropriate fluorophore‐conjugated secondary antibodies at room temperature in the dark. Nuclei were subsequently counterstained with DAPI, and the slides were coverslipped. Finally, fluorescence images were captured using a SOPTOP CX40 microscope and analyzed to assess the co‐localization and signal intensities of CASP8, ASC, and RIPK3. Image acquisition settings (exposure time, gain, and magnification) were kept consistent across all groups. At least three random fields per section were analyzed. Quantification was performed using ImageJ.

KGN cells were grouped and treated as described in the LDH assay (five groups, three replicates each). After 48 h of treatment, cells were seeded on coverslips, rinsed with PBS, and fixed with 4% paraformaldehyde. Subsequent procedures were performed as described above to detect the co‐localization of ASC, Caspase‐8, and RIPK3. Image acquisition and quantification for cell samples were performed under the same conditions.

### 2.13 Western Blot Analysis

Ovarian tissue samples (*n* = 3) were briefly rinsed with ice‐cold PBS, followed by homogenization in RIPA buffer containing inhibitors on ice for 30 min. Protein concentrations were determined by the BCA assay, followed by separation of equal amounts through SDS‐PAGE and electrophoretic transfer to PVDF membranes. Following blocking, membranes were incubated overnight at 4°C with primary antibodies: anti‐ZBP1 (1:1000), anti‐c‐Caspase‐3 (1:8000), anti‐Caspase‐3 (1:1000), anti‐GSDMD/N‐GSDMD (1:1000), anti‐GSDME (1:1000), anti‐MLKL (1:5000), anti‐p‐MLKL (1:500), and anti‐GAPDH (1:5000). After incubation with HRP‐conjugated secondary antibodies at room temperature, protein bands were visualized via chemiluminescence, followed by densitometric quantification using Image‐Pro Plus 6.0 (Media Cybernetics, USA).

KGN cells were grouped and treated as described in the LDH assay (five groups, four replicates each). After 48 h of treatment, cells were lysed and total protein was extracted. Subsequent procedures and quantification methods were identical to those described above.

### 2.14 Statistical Analysis

For animal experiments, each rat was considered an independent biological replicate (*n* = 6 per group for functional and histological assays; *n* = 3 for Western blot and immunofluorescence). For cell experiments, three to six independent technical replicates were performed. For statistical evaluation, IBM SPSS Statistics (version 26.0) and GraphPad Prism (version 10.4.1) were used. Results were expressed as mean ± standard error of the mean (SEM). Normality was assessed using the Shapiro–Wilk test, and homogeneity of variances was evaluated using Levene's test. For data meeting both normality and homoscedasticity assumptions, one‐way ANOVA followed by Tukey's post hoc test was applied. For data that violated these assumptions, the Kruskal–Wallis *H* test followed by Dunn's post hoc test was used. Statistical significance was set at *p* < 0.05.

## 3 Results

### 3.1 TSG Ameliorates Ovarian Function in POI Rats

**FIGURE 1:**
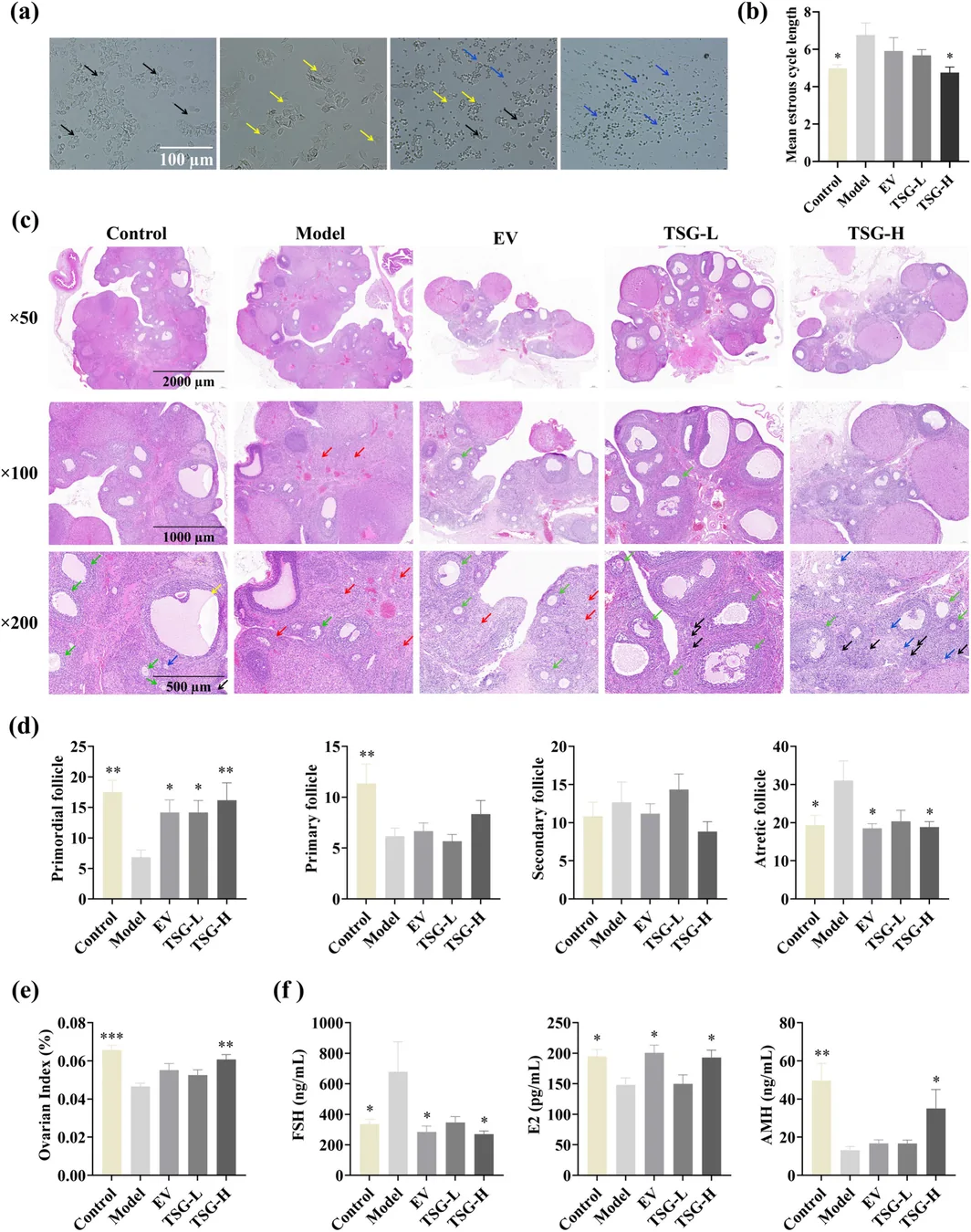
TSG ameliorates ovarian function in POI rats. (a) Representative vaginal cytology images of estrous cycles. Arrows indicate: Nucleated epithelial cells (black), cornified epithelial cells (yellow), and leukocytes (blue). (b) Mean estrous cycle length. (c) H&E‐stained ovarian sections. Arrows indicate: Primordial (black), primary (blue), secondary (green), and atretic (red) follicles. (d) Follicle counts. (e) Ovarian index. (f) Serum FSH, E_2_, and AMH levels. Mean ± SEM (*n* = 6). *p < 0.05, **p < 0.01, ***p < 0.001 versus model.

Normal rats exhibit regular 4–5‐day estrous cycles, with vaginal cytology revealing characteristic cellular morphologies across its phases: proestrus (predominantly nucleated epithelial cells), estrus (numerous keratinized epithelial cells), metestrus (a mixture of keratinized and nucleated epithelial cells along with leukocytes), and diestrus (predominantly leukocytes) (Shreya et al. 2025) (Figure 1a). During the 28‐day treatment period, POI model rats mainly displayed metestrus or diestrus phases, resulting in significantly extended mean estrous cycle length compared to control rats. Mean estrous cycle length was calculated as 28 days divided by the number of complete cycles observed during the treatment period. TSG‐H treatment effectively shortened the cycle length to the normal range (Figure 1b). Excessive follicle atresia and primordial follicle loss are central to POI pathogenesis (Spears et al. 2019). H&E staining revealed preserved ovarian cortex structure with abundant primordial follicles in control rats, while POI rats showed significantly fewer primordial follicles and a higher number of atretic follicles. EV and TSG treatments preserved primordial follicles and reduced atresia, suggesting protective efficacy against POI (Figure 1c,d). Additionally, the ovarian index was significantly reduced in POI rats, while TSG‐H treatment restored it (Figure 1e). Serum levels of AMH, FSH, and E_2_ reflect ovarian reserve (Panay et al. 2024). Compared to controls, POI model rats showed reduced AMH and E_2_ and elevated FSH, confirming successful modeling. Both EV and TSG‐H reversed the FSH and E_2_ changes, and TSG‐H additionally increased AMH levels (Figure 1f). These results indicate that EV and TSG effectively ameliorate ovarian function in POI rats.

### 3.2 TSG Treatment Is Associated With Reduced PANoptosis in POI Rats

**FIGURE 2:**
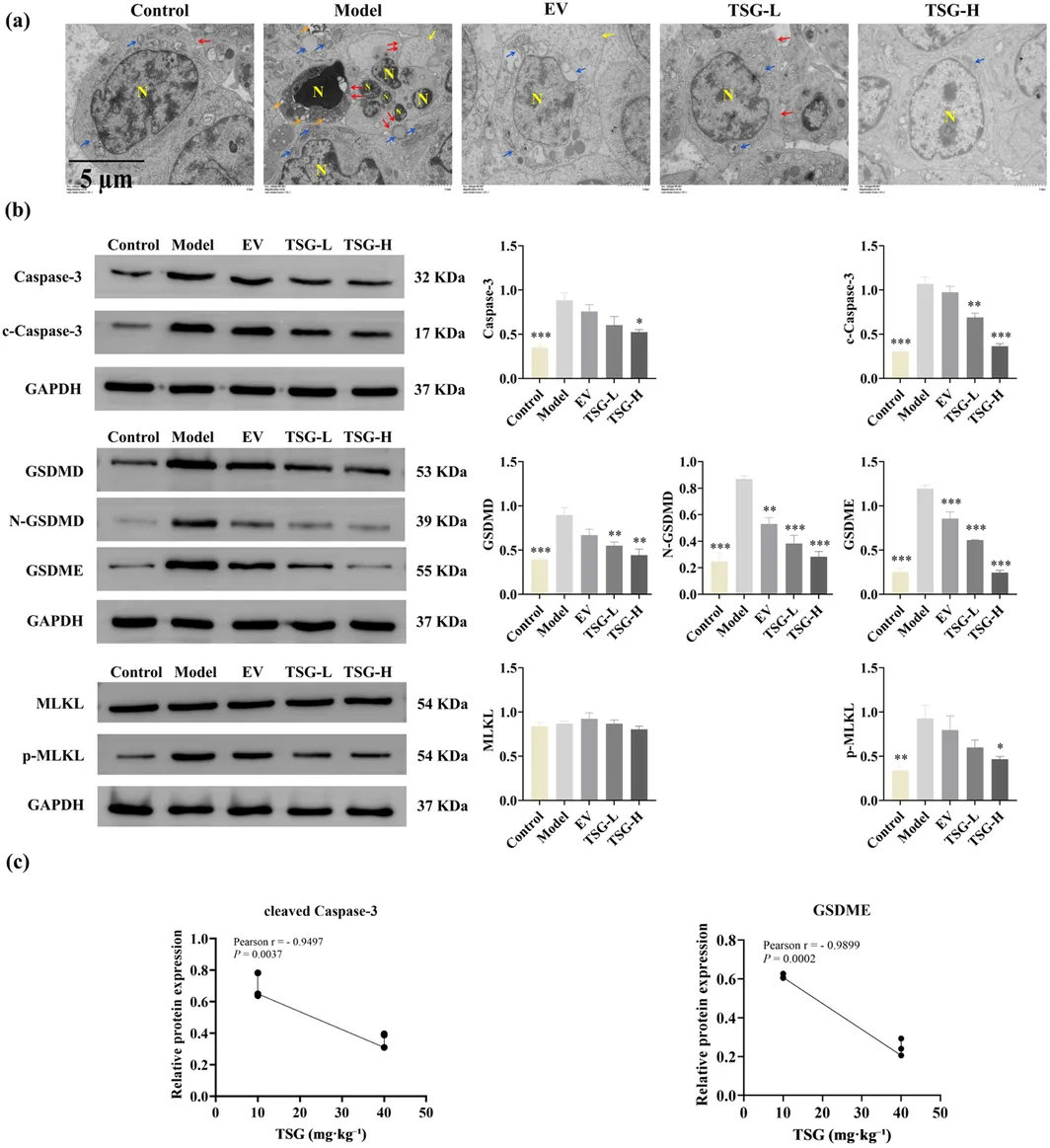
TSG treatment is associated with reduced panoptosis in POI rats. (a) TEM images of ovarian granulosa cells. N, nucleus; Yellow arrowheads, apoptotic bodies; Red arrowheads, membrane pore formation and rupture; Blue arrowheads, swollen organelles (mitochondria and endoplasmic reticulum); Orange arrowheads, cytoplasmic vacuolization. (b) Western blot analysis of panoptosis executioners. (c) Pearson correlation analysis between TSG doses (10 and 40 mg·kg^−1^) and the expression levels of cleaved Caspase‐3 (*r* = −0.9497, *p* < 0.01) and GSDME (*r* = −0.9899, *p* < 0.001). Mean ± SEM (*n* = 3). *p < 0.05, **p < 0.01, ***p < 0.001 versus model.

Granulosa cells constitute the largest cell population within ovarian follicles and critically regulate follicular development, maturation, and atresia (Liu, Jia, et al. 2023). TEM revealed normal ultrastructure in control granulosa cells. In contrast, the model group exhibited severe cellular damage, including nuclear pyknosis and fragmentation, organelle swelling, cytoplasmic vacuolization, and plasma membrane rupture with cytoplasmic content spillage, hallmarks indicative of concurrent pyroptosis, apoptosis, and necroptosis, consistent with PANoptosis‐like cell death. Pharmacological intervention was markedly associated with improvement of these ultrastructural lesions, with the TSG‐H group showing near‐normal granulosa cell ultrastructure, with only occasional organelle swelling and minor membrane disruptions (Figure 2a).

Western blot analysis was used to examine the PANoptosis executioners (Figure 2b). Compared to controls, the model group exhibited significantly elevated levels of apoptosis‐related proteins Caspase‐3 and c‐Caspase‐3, pyroptosis execution proteins GSDMD, N‐GSDMD, and GSDME, and necroptosis protein p‐MLKL. This suggests the concurrent activation of pyroptotic, apoptotic, and necroptotic pathways, indicative of PANoptosis induction in the ovaries of POI model rats. TSG treatment significantly reduced their expression. Notably, EV treatment successfully reduced N‐GSDMD and GSDME expression, although its suppressive effect was less pronounced compared to the TSG intervention groups. In summary, protein‐level alterations align with the ultrastructural enhancements seen under TEM, together validating TSG's superior effectiveness in suppressing PANoptosis. Pearson correlation analysis further revealed that TSG dose was significantly negatively correlated with the expression of cleaved Caspase‐3 and GSDME (Figure 2c), supporting the association between TSG treatment and reduced PANoptosis effector expression.

### 3.3 TSG‐Treated Rats Showed Reduced ZBP1/PANoptosome Formation

**FIGURE 3:**
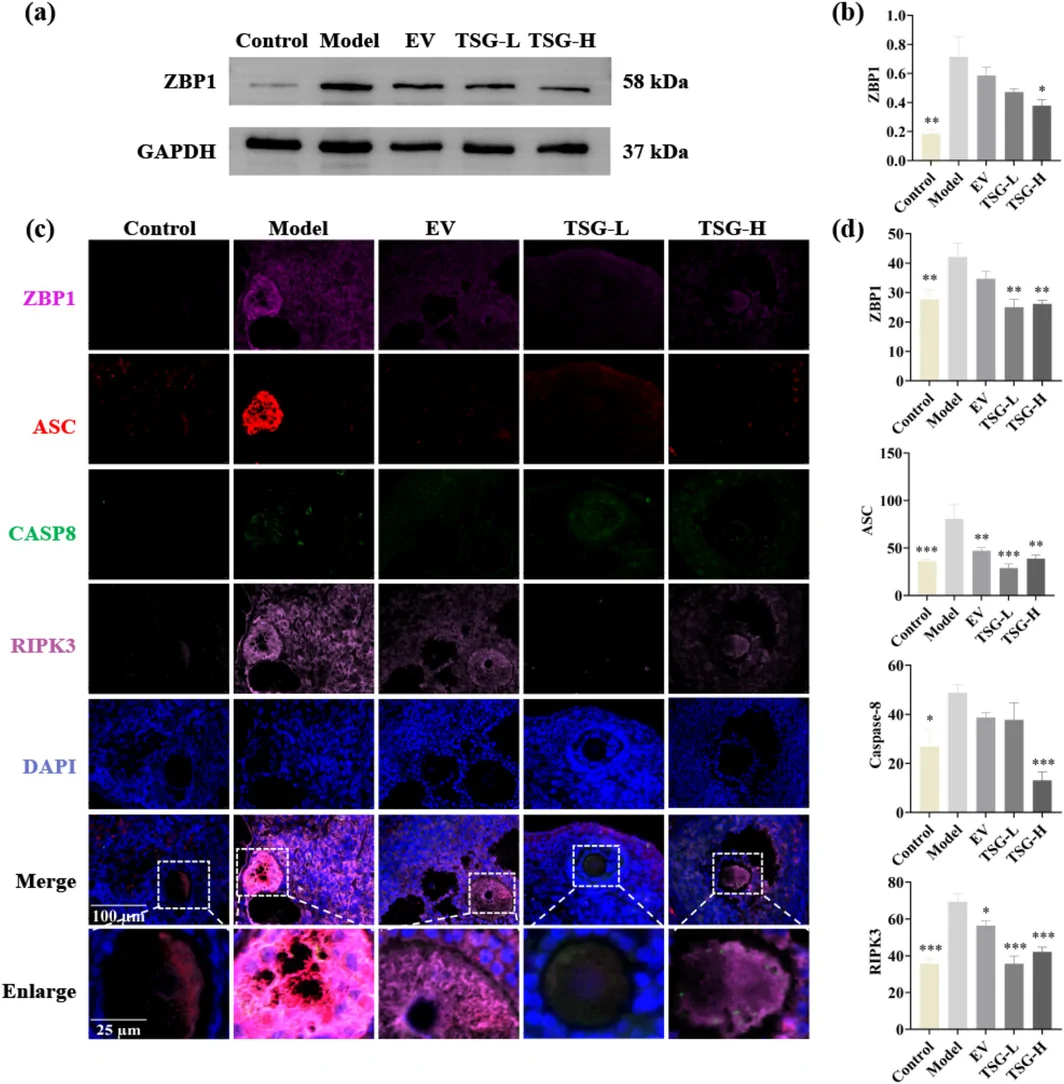
TSG‐treated rats showed reduced ZBP1/PANoptosome formation in POI rats. (a, b) Western blot analysis of ZBP1 levels. (c) Immunofluorescence co‐localization of ZBP1 (magenta), ASC (red), Caspase‐8 (green), and RIPK3 (pink) in follicles. (d) Average fluorescence intensity. Mean ± SEM (*n* = 3). *p < 0.05, **p < 0.01, ***p < 0.001 versus model.

ZBP1 is an upstream key molecule of PANoptosis (Karki et al. 2021). Western blot analysis revealed a significant increase in ZBP1 expression in the POI rats compared to the controls. This elevated level was markedly reduced following TSG‐H intervention (Figure 3a,b).

PANoptosis is governed by the PANoptosome, an intricate multiprotein complex incorporating critical elements like ASC, Caspase‐8, and RIPK3, commonly identified through immunofluorescence co‐localization (You et al. 2025; Xie et al. 2024). Immunofluorescence staining in our study revealed low ZBP1, ASC, Caspase‐8, and RIPK3 expression with no clear co‐localization in follicles from control rats. In contrast, POI model rats displayed striking co‐localization—especially in granulosa cells of follicles—along with markedly elevated fluorescence intensities compared to controls. This indicates the formation of the ZBP1/PANoptosome, which is associated with subsequent PANoptosis and excessive follicular atresia in POI rats. Both EV and TSG treatments diminished this co‐localization, with TSG‐H markedly lowering the fluorescence intensity of ZBP1, ASC, Caspase‐8, and RIPK3 (Figure 3c,d). These observations indicate that EV and TSG treatment are associated with reduced ZBP1/PANoptosome formation and diminished PANoptosis in POI ovaries, correlating with mitigation of POI; among these, TSG‐H showed the most pronounced effect.

### 3.4 Establishment of Cellular POI Model and TSG Dose Screening

**FIGURE 4:**
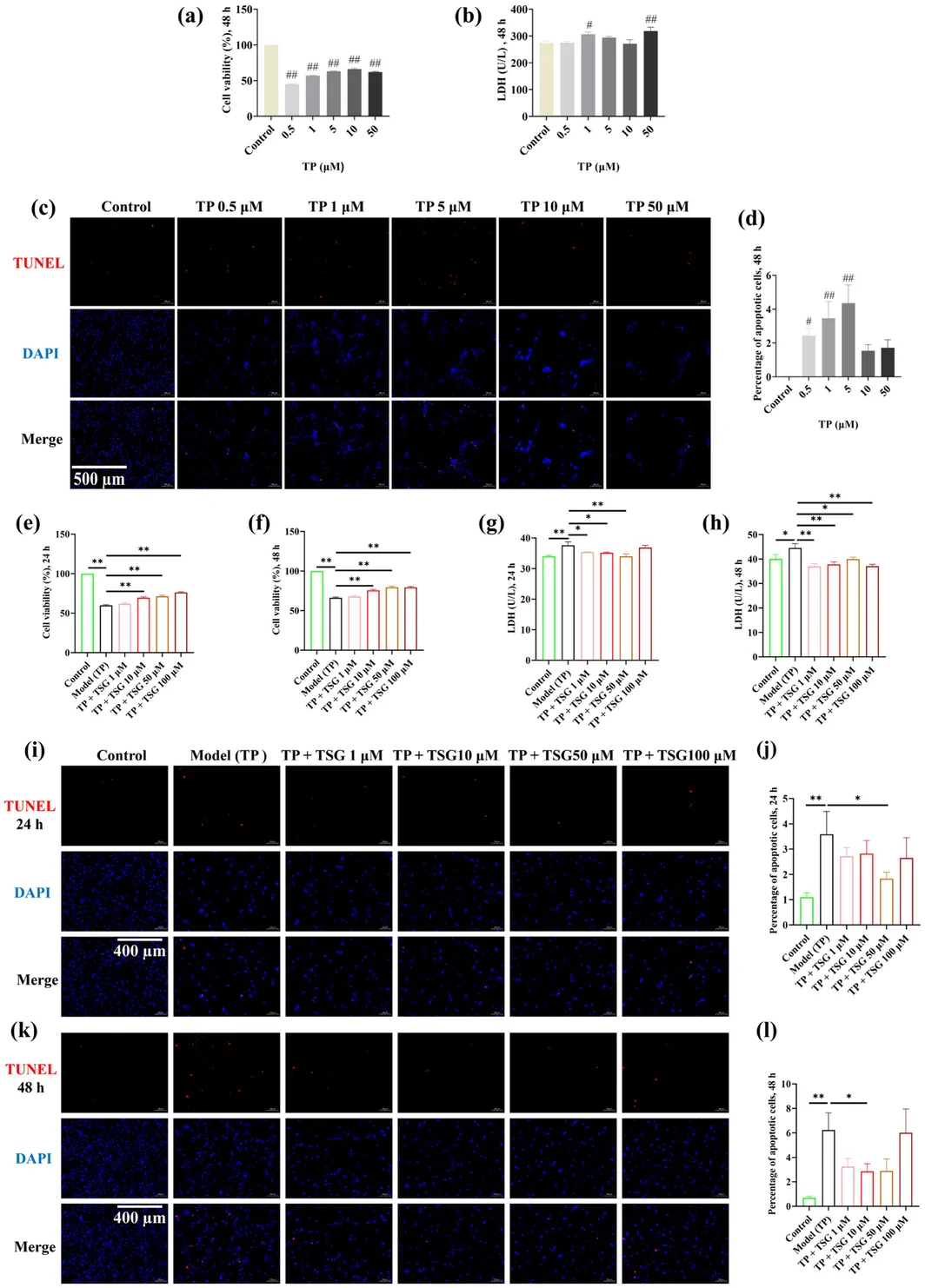
Establishment of cellular POI model and TSG dose screening in KGN cells. TP optimization using (a) cell viability (CCK‐8 assay), (b) lytic cell death (LDH release assay), and (c–d) apoptosis (TUNEL staining). TSG optimization using (e–f) cell viability (CCK‐8 assay), (g–h) lytic cell death (LDH release assay), and (i–l) apoptosis (TUNEL staining). Mean ± SEM (*n* = 3–6). ^#^ *p* < 0.05, ^##^ *p <* 0.01 vs. control. *p < 0.05, **p < 0.01 versus model.

Excessive death of granulosa cells leads to accelerated follicle atresia and ovarian functional decline (Liu, Chen, et al. 2023). To induce the cellular POI model, the granulosa cell KGN cells were incubated with TP (Gao et al. 2025; Zhu, Yao, et al. 2024), and the optimal concentration was evaluated via the CCK‐8 assay, LDH release assay, and TUNEL staining. The findings (Figure 4a–d) indicated that TP treatment at 1 μM for 48 h triggered considerable cellular damage, rendering this concentration appropriate for creating an in vitro POI model in subsequent experiments. The screening results (Figure 4e–i) revealed that TSG significantly attenuated TP‐induced injury under two regimens: 50 μM for 24 h, or 10 μM for 48 h. Given the POI model established via 48 h TP treatment, 10 μM TSG (co‐administered for 48 h) was selected to evaluate its potential protective and therapeutic effects in follow‐up experiments.

### 3.5 TSG Rescues the Aggravated Cellular Injury Induced by Oe‐ZBP1

**FIGURE 5:**
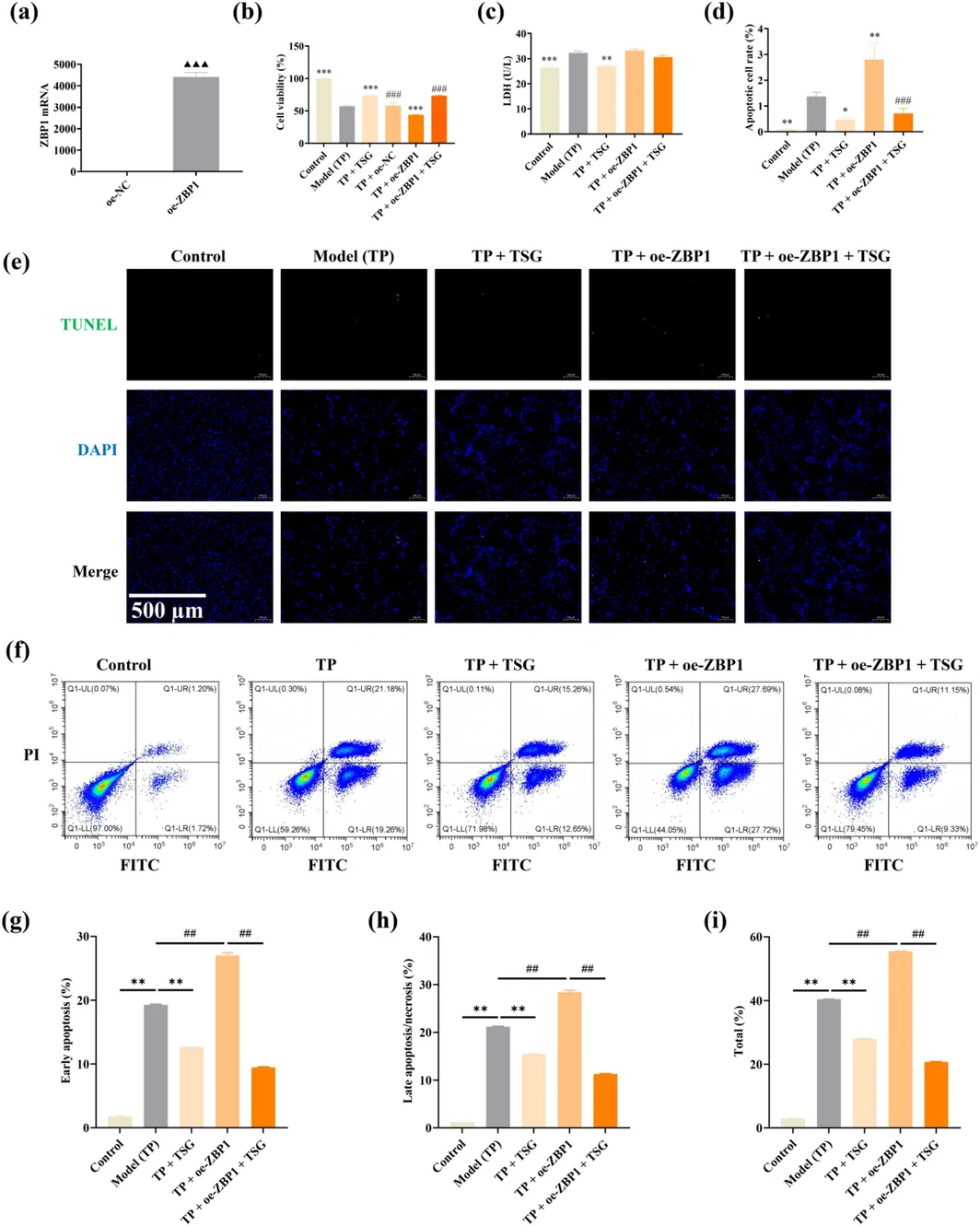
TSG rescues the aggravated cellular injury induced by oe‐ZBP1 in cellular POI model. (a) Validation of ZBP1 overexpression (qPCR). (b) Cell viability (CCK‐8 assay). (c) Lytic cell death (LDH release assay). (d–e) Apoptosis detection (TUNEL staining; green, TUNEL‐positive cells; blue, DAPI). (f–i) Apoptosis/necrosis analysis (Annexin V‐FITC/PI staining). Mean ± SEM (*n* = 3–5). ^▲▲▲^ *p* < 0.001 versus oe‐NC group. *p < 0.05, **p < 0.01, ***p < 0.001 versus model group. ##*p <* 0.01, ^###^ *p <* 0.001 versus TP + oe‐ZBP1 group.

To examine whether TSG exerts its effects in association with ZBP1/PANoptosis, we overexpressed ZBP1 (oe‐ZBP1) in the cellular POI model and evaluated the resulting cellular damage along with TSG's protective effects. qPCR data (Figure 5a) confirmed successful overexpression. Regarding cellular damage, the model (TP) group significantly reduced cell viability (Figure 5b), increased LDH release (Figure 5c), and increased apoptotic/necrotic rates (Figure 5d–i) compared to control. TSG intervention (TP + TSG group) was significantly associated with amelioration of these damage indicators. Transfection with oe‐NC under TP intervention did not alter cell viability compared to the TP model group. Notably, oe‐ZBP1 exacerbated TP‐induced cellular injury, an effect that was rescued by TSG co‐treatment.

### 3.6 TSG Disrupts Oe‐ZBP1‐Facilitated PANoptosome Formation

**FIGURE 6:**
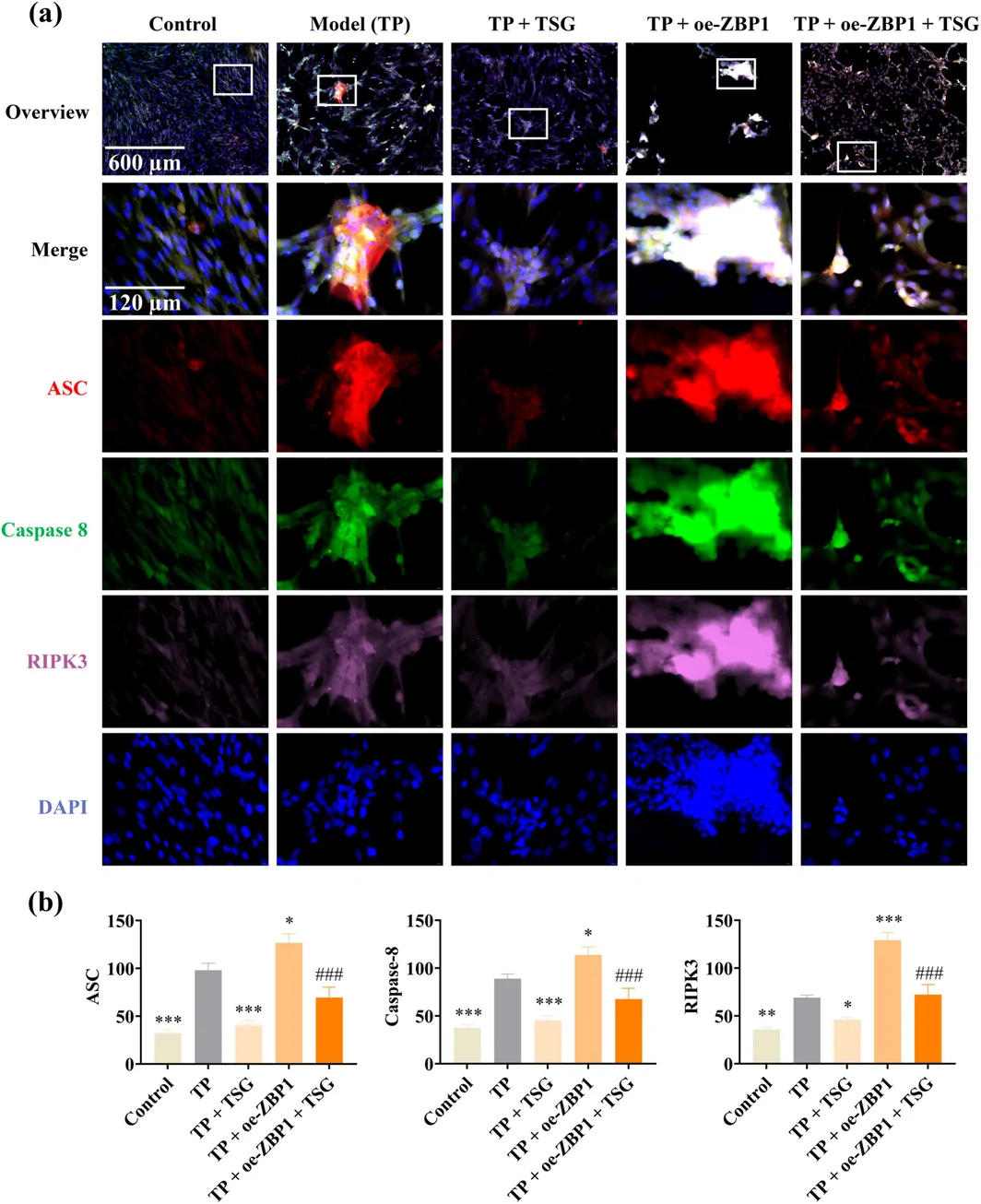
TSG treatment is associated with reduced oe‐ZBP1‐aggravated PANoptosome formation in cellular POI model. (a) Immunofluorescence co‐localization of ASC (red), Caspase‐8 (green), and RIPK3 (pink). (b) Average fluorescence intensity. Mean ± SEM (*n* = 3–5). *p < 0.05, **p < 0.01, ***p < 0.001 versus Model group; #*p <* 0.05, ^##^ *p* < 0.01, ^###^ *p* < 0.001 versus TP + oe‐ZBP1 group.

Building on evidence that oe‐ZBP1 exacerbates POI‐associated injury, we next examined its effect on PANoptosome formation and TSG intervention. Immunofluorescence co‐localization was used to observe the PANoptosome (You et al. 2025; Xie et al. 2024). Our results (Figure 6) showed that control cells formed a confluent monolayer with minimal expression and no evident co‐localization of Caspase‐8, ASC, and RIPK3. In TP‐induced (POI model) cells, reduced density was accompanied by strong co‐localization of these components; their fluorescence intensities were significantly higher than in controls, indicating active PANoptosome formation linked to excessive granulosa cell death. TSG co‐treatment markedly reduced both co‐localization and fluorescence intensity relative to the TP group. Furthermore, ZBP1 overexpression (oe‐ZBP1) exacerbated cellular injury and markedly elevated PANoptosome co‐localization as well as fluorescence intensity compared to TP treatment alone. Importantly, TSG co‐administration substantially reversed these pro‐injury effects. These results suggest that TSG may modulate this pathway in association with ZBP1, which is associated with reduced PANoptosome formation and amelioration of POI.

### 3.7 TSG Attenuates Oe‐ZBP1‐Aggravated PANoptosis

**FIGURE 7:**
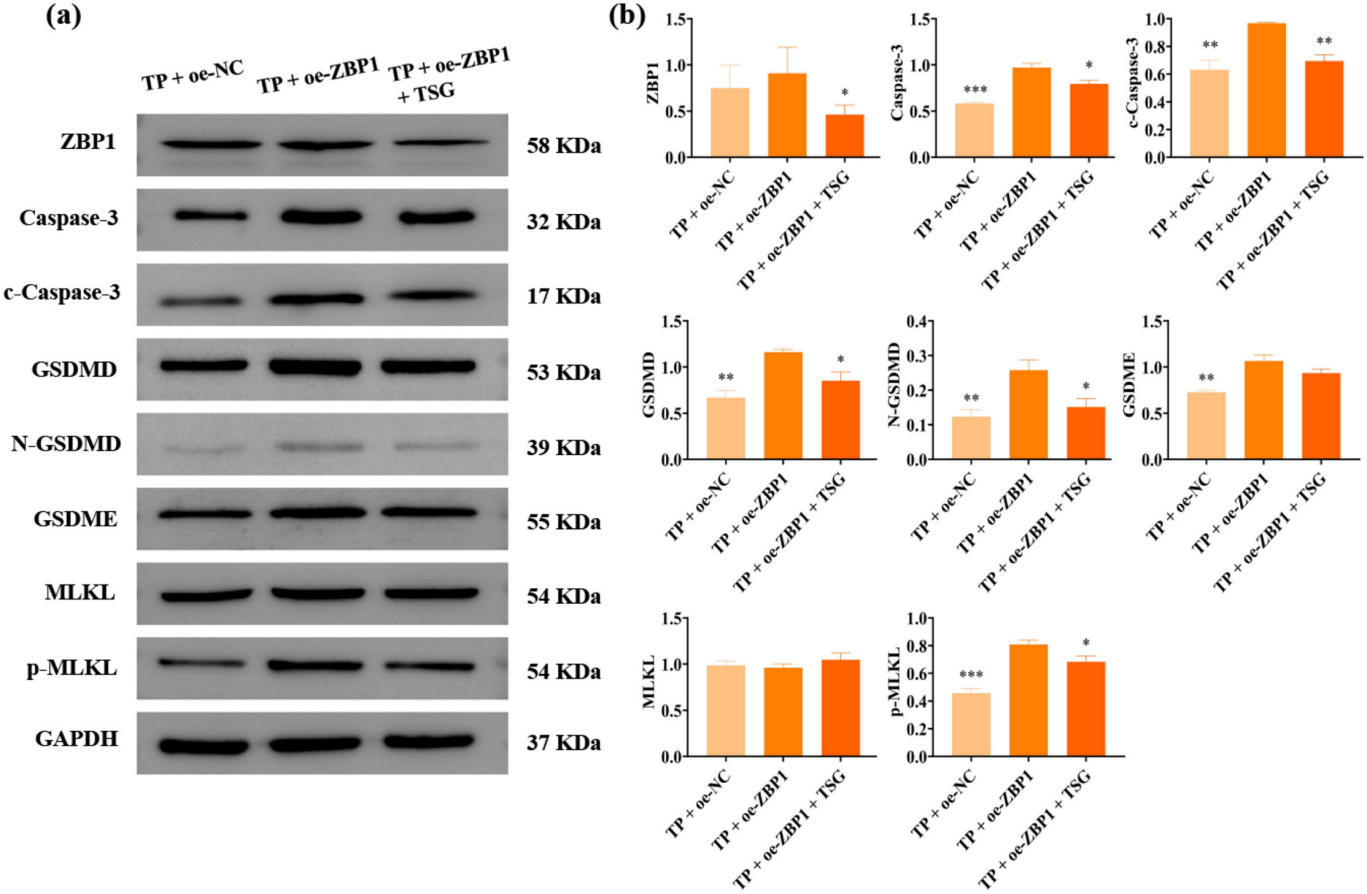
TSG attenuates oe‐ZBP1‐aggravated PANoptosis in cellular POI model. (a) Western blot analysis of PANoptosis‐related proteins. (b) Quantitative analysis. Mean ± SEM (*n* = 4). *p < 0.05, **p < 0.01, ***p < 0.001 versus TP + oe‐ZBP1 group.

Finally, we examined the inhibitory effect of TSG on PANoptosis executioners driven by ZBP1 overexpression. Western blot analysis (Figure 7) showed that, compared with TP + oe‐NC, TP + oe‐ZBP1 significantly increased levels of Caspase‐3, c‐Caspase‐3, GSDMD, N‐GSDMD, GSDME, and p‐MLKL. These results indicate that ZBP1 overexpression acts as a key upstream activator of the PANoptosis phenotype in POI model cells. Furthermore, TSG was associated with reversal of these changes, suggesting that TSG may modulate ZBP1/PANoptosis activation, which may be associated with the amelioration of POI.

## 4 Discussion

**FIGURE 8:**
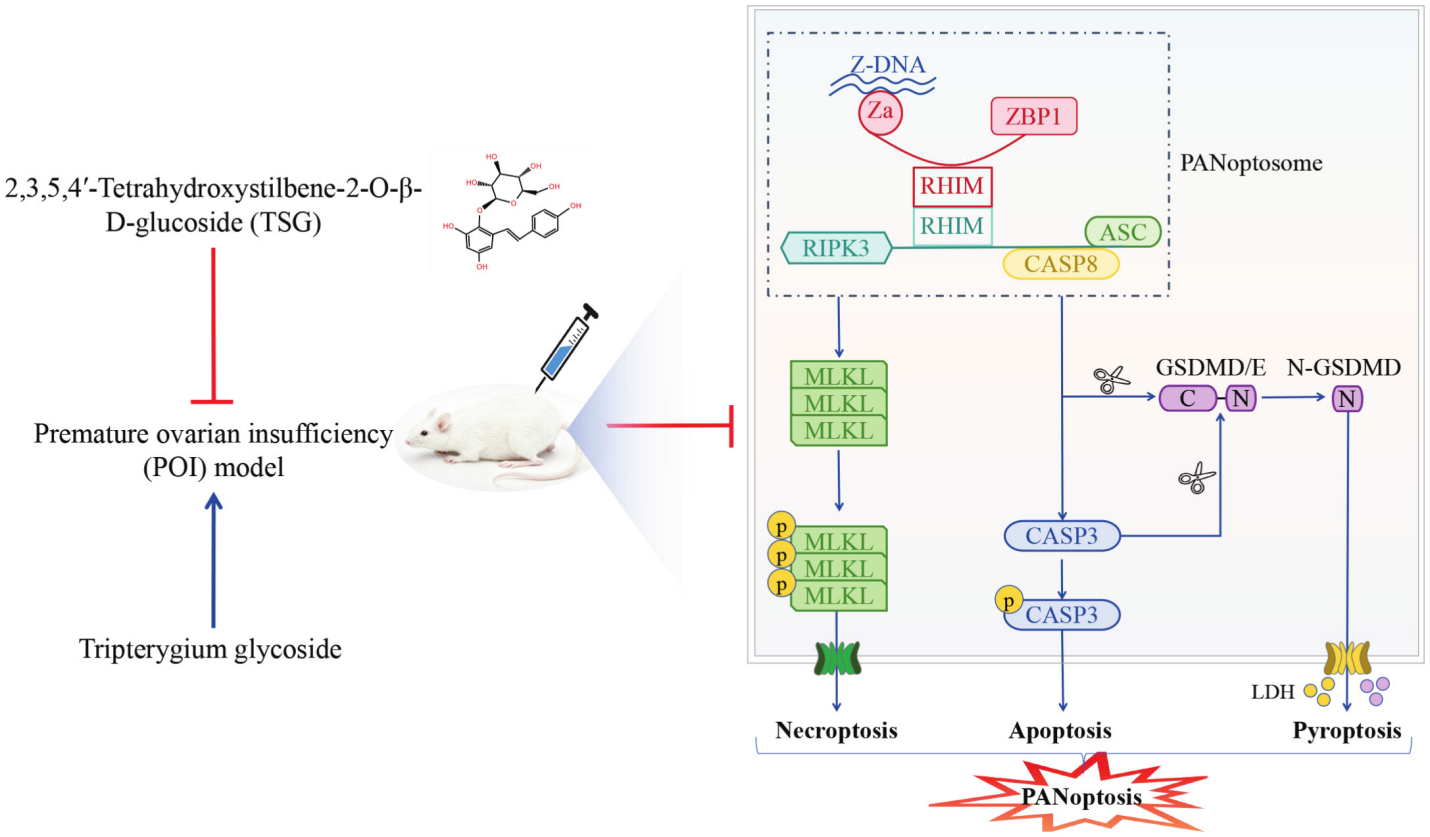
TSG treatment was associated with reduced ZBP1/PANoptosome formation and PANoptosis, which were associated with the amelioration of POI.

Our recent study showed that the crude extract of *Polygonum multiflorum* Thunb. suppresses PANoptosis and alleviates POI (Zhu, Li, Li, et al. 2025), but did not identify the responsible bioactive constituent or examine upstream regulation of PANoptosis. Here, we extend those findings by demonstrating that TSG, the principal active component of this plant, correlates with suppression of ZBP1/PANoptosis in vivo and in vitro, advancing the mechanistic understanding from a crude extract to a defined compound and from phenotypic observation to pathway‐focused investigation. Tripterygium glycosides and triptolide are derived from the same plant source, *Tripterygium wilfordii* Hook.F. Both agents induce ovarian injury through shared mechanisms, supporting their use as complementary models for investigating POI (Xiao et al. 2026). Our findings demonstrate that TSG substantially ameliorates ovarian dysfunction in a tripterygium glycoside‐induced POI rat model and attenuates triptolide‐induced injury in human KGN granulosa cells. Mechanistically, TSG exerts its protective effects in association with reduced ZBP1/PANoptosome formation and subsequent PANoptosis activation, thereby reducing extensive follicular atresia and ameliorating POI (Figure 8).

A regular estrous cycle is a vital indicator of female reproductive health (Liu, Shi, et al. 2024), while the irreversible depletion of primordial follicles, the fundamental functional units of the ovarian reserve, is considered the core pathological basis of POI (He et al. 2021). In this study, TSG administration was associated with restored estrous cycle regularity and the ovarian index in POI rats, while significantly curtailing excessive primordial follicle depletion and follicular atresia, thus preserving ovarian reserve. Regarding hormonal profiles, serum FSH, E_2_, and AMH are key biochemical markers for POI diagnosis (Panay et al. 2024). TSG was associated with improved hormonal profiles in POI rats, indicating its potential to restore ovarian endocrine function. From a cytomorphological perspective, granulosa cells in the POI model group exhibited severe ultrastructural damage, including apoptotic bodies and plasma membrane rupture, suggesting the coexistence of multiple cell death modalities such as pyroptosis, apoptosis, and necroptosis. TSG intervention markedly alleviated these morphological injuries, likely attributable to suppression of this mixed cell death pattern.

Further mechanistic studies showed that hallmark effector proteins of pyroptosis, apoptosis, and necroptosis were all upregulated in the POI model, and TSG was associated with their downregulation. PANoptosis is an inflammatory programmed cell death pathway mediated by ZBP1, which integrates multiple death cascades via the PANoptosome. We propose that TSG maintains ovarian homeostasis through mechanisms that may involve this key upstream sensor, ZBP1, with a consequent reduction in PANoptosis activation (Christgen et al. 2020; Karki et al. 2021; Zheng and Kanneganti 2020). Accordingly, we further examined ZBP1 (Lee et al. 2021). The results revealed that ZBP1 expression was elevated in POI conditions, promoting PANoptosome formation (comprising ASC, caspase‐8, and RIPK3), while TSG treatment correlated with downregulation of ZBP1 and reduced assembly of this complex. This discovery specifically connects TSG's ovarian protective action from downstream effector processes to an upstream signaling nexus. As the core initiator of PANoptosis, ZBP1 activation is a prerequisite for downstream coordinated cell death (Lee et al. 2021). One possible mechanism is that TSG may affect ZBP1‐related signaling, which may contribute to reduced PANoptosome formation and the attenuation of the synergistic effects of apoptosis, pyroptosis, and necroptosis.

Granulosa cells, essential somatic cells surrounding the oocyte, play pivotal roles in follicular development, steroid hormone synthesis, and oocyte maturation (Liu, Jia, et al. 2023; Sreerangaraja Urs et al. 2020). To establish a human cellular model of POI, granulosa cell injury was induced in vitro. This method progressed the research from whole‐animal models to a human cell line system, while streamlining the experimental design from intricate in vivo conditions to defined in vitro settings, thereby allowing direct confirmation of ZBP1's role in POI pathogenesis. After comprehensive assessment, 1 μM TP was selected to replicate granulosa cell injury in POI (Bai et al. 2019). Experimental data suggested that TSG effectively attenuated the TP‐induced reduction in cell viability and increase in apoptosis, confirming its potent protective effect in granulosa cells. To further investigate the mechanism of TSG, ZBP1 was overexpressed in the model cells. High ZBP1 expression significantly exacerbated TP‐induced cellular damage, manifesting as a further decline in viability and increased apoptosis, a phenomenon consistent with the functional characteristics of core PANoptosis regulatory proteins (Sun et al. 2024; Wang et al. 2024). Even under this condition, TSG administration was still associated with improved cell viability and reduced apoptosis. At the molecular assembly level, dense co‐localization of ASC, Caspase‐8, and RIPK3 was observed in the POI model, indicating PANoptosis activation in granulosa cells (Lee et al. 2021). TSG treatment was associated with reduced assembly of this complex, suggesting a possible link between its protective effect and PANoptosis inhibition. Notably, PANoptosome formation was further enhanced under ZBP1 overexpression, establishing ZBP1 as an upstream “trigger” that directly drives downstream complex formation (Ren et al. 2025). Importantly, TSG was still strongly associated with reduced complex aggregation under this condition, suggesting that TSG may affect ZBP1‐associated pathways; however, whether this involves direct interaction, activation, or interaction with other components remains to be investigated. At the protein execution level, ZBP1 overexpression concurrently upregulated key execution proteins of apoptosis (Caspase‐3/c‐Caspase‐3), pyroptosis (GSDMD/N‐GSDMD, GSDME), and necroptosis (p‐MLKL), confirming ZBP1's role as an upstream initiator in the PANoptosis network capable of coordinating multiple cell death pathways. Under high ZBP1 expression conditions, TSG substantially reduced ZBP1 levels as well as the previously noted activated cell death effectors, suggesting a potential role for TSG in counteracting ZBP1/PANoptosis.

In addition to its association with ZBP1/PANoptosis modulation, TSG has been reported to possess phytoestrogenic activity. As summarized in our recent systematic review (Zhu, Li, Tang, et al. 2025), TSG promotes the proliferation of estrogen receptor‐positive cells and upregulates the expression of estrogen‐dependent genes. These estrogenic properties may represent an alternative or complementary mechanism underlying the ovarian protective effects of TSG, independent of PANoptosis. Furthermore, given that PANoptosis is an inflammatory form of programmed cell death, the anti‐inflammatory properties of TSG, as widely documented in the literature, may also contribute to the observed suppression of PANoptosis and subsequent ovarian protection. However, the relative contribution of these pathways remains to be elucidated in future studies.

Several limitations of this study should be acknowledged. First, while our data demonstrate a strong correlation between TSG‐mediated POI improvement and reduced ZBP1/PANoptosis, the absence of ZBP1 loss‐of‐function and co‐immunoprecipitation assays means causality has not been fully established, and whether ZBP1 is a direct target of TSG remains to be determined. In addition, the KGN cells, a granulosa‐like tumor cell line, may not fully represent primary granulosa cell physiology. Second, the administration protocol was primarily preventive/co‐treatment rather than therapeutic, as TSG was given concurrently with the POI‐inducing agent; accordingly, this study was designed to mainly assess prevention rather than treatment. Third, while the intraperitoneal route was chosen for mechanistic validation to ensure consistent absorption (Liu, Chen, et al. 2024), it differs from the oral route relevant to dietary use. Notably, intraperitoneal administration is widely accepted in mechanistic studies of dietary plant extracts, as the route can be flexibly tailored to the research objectives (Alkhathami et al. 2025; El‐Fakharany et al. 2025). Although oral TSG has shown beneficial effects on ovarian function (Lin et al. 2022), whether the doses used here are achievable through dietary or supplement consumption remains to be determined. Additionally, the oral bioavailability of TSG is limited by extensive intestinal and hepatic metabolism, and potential food‐matrix effects were not systematically examined in this study. Future studies employing oral administration combined with pharmacokinetic–pharmacodynamic analysis, along with systematic evaluation of dose achievability, food‐matrix effects, and functional fertility outcomes, will be valuable to bridge these mechanistic findings with translational and nutritional applications. Despite these limitations, the present study provides a solid foundation for understanding the role of ZBP1/PANoptosis in POI and positions TSG as a promising candidate for further translational investigation.

Regarding safety, although *Polygonum multiflorum* Thunb. has documented hepatotoxicity, particularly with raw material and at high doses (Min et al. 2025), and tripterygium glycosides are also known to have potential toxicity, no overt signs of toxicity (e.g., abnormal behavior, significant body weight loss, or mortality) were observed in any group throughout the experimental period. The TSG doses used (10 and 40 mg·kg^−1^) are within the reported safe range (Jiang et al. 2025; Liu, Shi, et al. 2024). Nevertheless, systematic safety assessment, including liver and kidney histopathology and serum biochemistry, was beyond the scope of this study and warrants further investigation.

In conclusion, our findings indicate that TSG can attenuate the development of POI in a rat model, in a manner associated with the suppression of ZBP1/PANoptosis. However, the present findings represent mechanistic evidence, further studies using oral administration and appropriate dose translation are required to explore the translational potential of TSG in dietary or supplement contexts.

## Author Contributions

**Jinhong Li:** methodology, writing – original draft, formal analysis, investigation. **Tiao Xie:** investigation. **Chang Lin:** conceptualization, data curation, writing – review and editing. **Danhong Peng:** conceptualization, data curation, writing – review and editing. **Qin Shi:** conceptualization, data curation, writing – review and editing. **Can Zhu:** conceptualization, data curation, writing – review and editing, supervision, funding acquisition.

## Funding

This research was funded by the National Natural Science Foundation of China (Grant No. 82360952) and the Guizhou University of Traditional Chinese Medicine Doctoral Research Initiation Fund (Grant No. GUCM‐DI [2024] 26).

## Disclosure

During the preparation of this work, the authors did not use any generative AI or AI‐assisted technologies for manuscript drafting, data analysis, figure generation, or literature review. All content was written and revised by the authors.

## Ethics Statement

The animal study protocol received approval from the Institutional Animal Ethics Committee at Guizhou University of Traditional Chinese Medicine (Approval No. 20241013001).

## Conflicts of Interest

The authors declare no conflicts of interest.

## Data Availability

The data that support the findings of this study are available from the corresponding author upon reasonable request.
